# Supplementary material for: Towards improving online learning in physical education: Gender differences and determinants of motivation, psychological needs satisfaction, and academic achievement in Saudi students
Source: PLoS One. 2024 Feb 6;19(2):e0297822. doi: 10.1371/journal.pone.0297822 (PMC10846739; doi:10.1371/journal.pone.0297822)
Supplement: S1 File — (DOCX) [file pone.0297822.s001.docx]

**S1 File. Physical Education Autonomy Relatedness Competence Scale (PEARCS Arabic version)**

**مقياس الكفاءة، التواصل والاستقلالية في التربية البدنية**

We are interested in your experiences in physical education (PE) class. Using the scale below, please indicate by circling, to what extent each of the following items is true for you. Please note that there are no right or wrong answers and no trick questions. We simply want to know how you personally feel about PE.

نحن مهتمون بتجربتك في مقرر التربية البدنية. باستخدام المقياس أدناه، يرجى الإشارة بالدائرة إلى أي مدى تنطبق عليك كل عبارة من العبارات التالية. يرجى ملاحظة أنه لا توجد إجابات صحيحة أو خاطئة ولا توجد أسئلة خادعة. نريد ببساطة أن نعرف كيف تشعر شخصيًا تجاه حصص التربية البدنية.

| أوافق بشدة | أوافق | محايد | لا أوافق | لا أوافق بشدة | When I am in PE... | أثناء حصص التربية البدنية... | |
| --- | --- | --- | --- | --- | --- | --- | --- |
| 5 4 3 2 1 | | | | | My classmates seem to like me | يبدو أن زملائي بالفصل معجبين بي | 1 |
| 5 4 3 2 1 | | | | | I am good at the things we do | أعتبر نفسي جيدا في الأشياء التي أقوم بها | 2 |
| 5 4 3 2 1 | | | | | I can choose which activities I want to practice | يمكنني اختيار الأنشطة التي أرغب في ممارستها | 3 |
| 5 4 3 2 1 | | | | | I really like the people I am with | أنا حقًا أتقبل زملائي بالفصل | 4 |
| 5 4 3 2 1 | | | | | I am able to perform well | أنا قادر على الأداء الجيد | 5 |
| 5 4 3 2 1 | | | | | I make a lot of my own decisions | أتخذ الكثير من القرارات الفردية | 6 |
| 5 4 3 2 1 | | | | | I feel like my classmates accept me | أشعر أن زملائي في الفصل يتقبلونني | 7 |
| 5 4 3 2 1 | | | | | I feel skilled | أشعر بقدرتي على أداء المهارات المطلوبة | 8 |
| 5 4 3 2 1 | | | | | I have input on which skills I want to practice | لدي دراية مسبقة بالمهارات التي أرغب بممارستها | 9 |
| 5 4 3 2 1 | | | | | I feel connected to my classmates | أشعر بالاندماج مع زملائي بالفصل | 10 |
| 5 4 3 2 1 | | | | | I am confident in my ability to learn | أنا واثق من قدرتي على التعلم | 11 |
| 5 4 3 2 1 | | | | | I am doing what I want | أتمتع بالحرية التامة | 12 |

Codification key: Autonomy items: Items 3, 6, 9, 12; Competence items: Items 2, 5, 8, 11; Relatedness items: Items 1, 4, 7, 10.
