# Supplementary material for: Towards improving online learning in physical education: Gender differences and determinants of motivation, psychological needs satisfaction, and academic achievement in Saudi students
Source: PLoS One. 2024 Feb 6;19(2):e0297822. doi: 10.1371/journal.pone.0297822 (PMC10846739; doi:10.1371/journal.pone.0297822)
Supplement: S2 File — (DOCX) [file pone.0297822.s002.docx]

**S2 File. Physical Education Motivation Scale (PEMS Arabic version)**

**مقياس التحفيز في التربية البدنية**

We are interested in your experiences in physical education (PE) class. Using the scale below, please indicate by circling, to what extent each of the following items is true for you. Please note that there are no right or wrong answers and no trick questions. We simply want to know how you personally feel about PE.

نحن مهتمون بتجربتك في مقرر التربية البدنية. باستخدام المقياس أدناه، يرجى الإشارة بالدائرة إلى أي مدى تنطبق عليك كل عبارة من العبارات التالية. يرجى ملاحظة أنه لا توجد إجابات صحيحة أو خاطئة ولا توجد أسئلة خادعة. نريد ببساطة أن نعرف كيف تشعر شخصيًا تجاه حصص التربية البدنية.

| أوافق بشدة | أوافق | محايد | لا أوافق | لا أوافق بشدة | When I am in PE... | أثناء حصص التربية البدنية... | |
| --- | --- | --- | --- | --- | --- | --- | --- |
| 5 4 3 2 1 | | | | | I participate in PE because it is fun | أشارك في حصص للتربية البدنية لأنها تشعرني بالمرح | 13 |
| 5 4 3 2 1 | | | | | I try to do well in PE so my teacher will think I am a good student | أحاول أن أبلي بلاءً حسناً لأظهر لأستاذي أنني طالب متميز | 14 |
| 5 4 3 2 1 | | | | | I don’t see the point of participating in PE | لا أرى فائدة من المشاركة خلال حصص التربية البدنية | 15 |
| 5 4 3 2 1 | | | | | I participate in PE because it is interesting | أشارك في حصص التربية البدنية لأنها مشوقة | 16 |
| 5 4 3 2 1 | | | | | I try hard in PE because I want a good grade | أحاول جاهداً في حصص التربية البدنية سعيا للحصول على درجات متميزة | 17 |
| 5 4 3 2 1 | | | | | I don’t see why I have to take PE | لا أرى أهمية لدراستي بمقرر التربية البدنية | 18 |
| 5 4 3 2 1 | | | | | I find PE enjoyable | أجد حصص التربية البدنية ممتعة | 19 |
| 5 4 3 2 1 | | | | | I do my best so my PE teacher will like me | أبذل قصارى جهدي لنيل اعجاب المدرس | 20 |
| 5 4 3 2 1 | | | | | PE is a waste of my time | أعتبر حصص التربية البدنية مضيعة لوقتي | 21 |

Codification key: Intrinsic motivation items: Items 13, 16, 19; Extrinsic motivation items: Items 14, 17, 20; Amotivation: Items 15, 18, 21
