# Supplementary material for: Towards improving online learning in physical education: Gender differences and determinants of motivation, psychological needs satisfaction, and academic achievement in Saudi students
Source: PLoS One. 2024 Feb 6;19(2):e0297822. doi: 10.1371/journal.pone.0297822 (PMC10846739; doi:10.1371/journal.pone.0297822)
Supplement: Attachment — Submitted filename: Responce to reviewers.docx [file pone.0297822.s003.docx]

To Professor **Chao Gu**

The Academic Editor	11/26/2023

**PLOS ONE**

Dear Editor,

All co-authors of the manuscript (ID: PONE-D-23-28306) entitled “*Towards improving online learning in physical education: gender differences and determinants of motivation, psychological needs satisfaction, and academic achievement in Saudi students*” and I, want to thank you for the consideration to our work. We agree with the comments advanced by the reviewers and believe that the requested rectifications can improve the quality of the manuscript.

Here you find the point-by-point comments and answers.

**Answers point by point to the Editor and reviewers' comments**

(Manuscript PONE-D-23-28306)

**Answers to the Editor's questions:**

| **Editor** | | |
| --- | --- | --- |
|  | **questions** | **answers** |
| **1** | 1. Please ensure that your manuscript meets PLOS ONE's style requirements, including those for file naming. | Style requirements verified. Changes in the headings and tables were made.  File naming verified. |
| **2** | We note that you have provided funding information that is not currently declared in your Funding Statement. However, funding information should not appear in the Acknowledgments section or other areas of your manuscript. We will only publish funding information present in the Funding Statement section of the online submission form. | The funding information was removed from the acknowledgment section.  Please insert this statement in the online submission form:  **Funding**: This study was funded by the Deanship  of Scientific Research at King Faisal University,  Saudi Arabia (grant number GRANT3961). The funder had no role in study design, data collection and analysis, decision to publish, or preparation of the manuscript. |
| **3** | We note that you have stated that you will provide repository information for your data at acceptance. Should your manuscript be accepted for publication, we will hold it until you provide the relevant accession numbers or DOIs necessary to access your data. If you wish to make changes to your Data Availability statement, please describe these changes in your cover letter and we will update your Data Availability statement to reflect the information you provide. | Data for the present are now published in Harvard Dataverse. Here is the DOI  <https://doi.org/10.7910/DVN/WQWLMU> |

**Answers to the reviewer 1 questions**

| **Reviewer 1** | | |
| --- | --- | --- |
|  | **questions** | **answers** |
| **1** | The title of the manuscript is informative and reads well. However, Authors could consider shortening the title of the manuscript. | To avoid any confusion in meaning, the title was reformed to "*Towards improving online learning in physical education: gender differences and determinants of motivation, psychological needs satisfaction, and academic achievement in Saudi students*". However, due to multiple study variables, its length was not reduced. |
| 2 | The authors could provide more suggestions for future research. Future research could rely on a specific classification system of motivational behaviors. | A study perspective was added in relation to the findings of Ahmadi et al (2023).  Lines 738-742: Moreover, the teachers’ in-class behavior is considered a key factor that influences students’ motivation [58]. Thus, promoting students' motivation during PE online learning according to an individualized specific classification system of teachers' motivational behaviors remains an interesting future research topic too [58]. |

**Answers to the reviewer 2 questions**

| **Reviewer 2** | | |
| --- | --- | --- |
|  | **questions** | **answers** |
| **1** | the title "Towards online physical education learning:" is confusing | To avoid any confusion in meaning, the title was reformed to "*Towards improving online learning in physical education: gender differences and determinants of motivation, psychological needs satisfaction, and academic achievement in Saudi students*". |
| **2** | The introduction needs some improvements: it should highlight the research gap with previous studies. Besides, it should status the objective of conducting the binary logistic regression. | The introduction was improved. Indeed a paragraph related to the Cultural and social characteristics of the Saudi population was added. (L95-102). The introduction was divided into three sections.  Logistic regression is an extension of “regular” linear regression, used when the dependent variable, Y, is categorical (non-metric). The binary logistic regression, in studies in which the dependent variable is a “Yes/No” type variable. Typically we refer to the two categories of Y as “1” and “0,” so that they are represented numerically. This is the case of the GPA variable where students were divided into a high GPA group (3 < GPA ≤ 5); and a low GPA group (1 < GPA ≤ 3). |
| **3** | The literature review and hypothesis sections are missing. The authors should provide a definition of each variable, especially the PNS components in the literature review section, and explain its association with the research topic. | The introduction was re-structured and the three study hypotheses were added at the end. |
| **4** | The results need to be further enhanced. It stressed an unequal samples t-test but did not reveal whether equal or unequal variance was assumed. | Welch's t-test also known as the unequal variances t-test is used when you want to test whether the means of two populations are equal. This test is generally applied when there is a difference between the variations of two populations and also when their sample sizes are unequal, which corresponds to our study.  Thus in the present study, equal variance was not assumed in all comparisons.  Changes are made in the result section accordingly. |
| **5** | The discussion in section 3.3 didn't explain the parenthesis parameters (the meaning of small, medium, and n square). | Explanation was added in the data analysis section. Partial eta squared is a way to measure the effect size. According to Cohen (1992) it is classified as small (0.1), medium (0.3), and large (> 0.5) |
| **6** | In section 3.4, the authors elaborate on the comparison between group and gender without supporting results or tables. The "experience to OL" in Table 4 should clarified as "prior experience with OL". | The results presented (L314-350) are additional results to better understand the detected differences between variables and show the variation of PNS and motivation according to the GPA in males only and in females only. However, we do not opt to present a table in order to avoid repetitions. If the reviewer believes that is necessary we can add a table related to those results. |
| **7** | The exp (B) values reported in Table 6 were wrong. For positive beta, the exp (B) values should be greater than 1; for negative beta (AM), the exp (B) value should be less than 1. | We fully agree with this remark. For negative B the Exp(B) should be less than 1 with is the case of amotivation variable. Indeed, by reviewing all the stat procedures and calculations, we discover mistakes in variable encoding generating errors in positive and negative beta values as well as in the Exp(B). However, the absolute beta values were correct.  Results of the binary LR are fully presented in the result section (L385-469). |
| **8** | Discussion: suggested the authors associate the findings with research objectives. | The discussion was revised and study limitations improved (L6955-698) (L739-742). |

**The corresponding author**

**Frikha Mohamed**
