# Supplementary material for: Towards improving online learning in physical education: Gender differences and determinants of motivation, psychological needs satisfaction, and academic achievement in Saudi students
Source: PLoS One. 2024 Feb 6;19(2):e0297822. doi: 10.1371/journal.pone.0297822 (PMC10846739; doi:10.1371/journal.pone.0297822)
Supplement: Attachment — Submitted filename: Response to Reviewers.docx [file pone.0297822.s004.docx]

To Professor **Chao Gu**

The Academic Editor	10/01/2024

**PLOS ONE**

Dear Editor,

All co-authors of the manuscript (ID: PONE-D-23-28306) entitled “*Towards improving online learning in physical education: gender differences and determinants of motivation, psychological needs satisfaction, and academic achievement in Saudi students*” and I, want to thank you for the consideration to our work. Here you find the point-by-point answers to your comments.

**Answers point by point to the Editor comments**

(Manuscript PONE-D-23-28306)

**(All changes in the manuscript are in red)**

| **Editor** | | |
| --- | --- | --- |
|  | **questions** | **answers** |
| **1** | Please further explain in Tables 2, 3, and 4 what the 95% confidence intervals (95% CI) correspond to in terms of values. | The 95% confidence interval defines a range of values that you can be 95% certain contains the population mean. In tables 2,3, and 4 the 95% confidence intervals correspond to the range of differences between means. Additional indications for CI were highlighted in the tables. |
| **2** | in the abstract, please narrate in a storytelling manner without including specific numerical details related to statistics. | Abstract was revised and numerical details were removed. |

**The corresponding author**
